# Prediction for recurrence following antithyroid drug therapy for Graves’ hyperthyroidism

**DOI:** 10.20945/2359-3997000000609

**Published:** 2023-05-10

**Authors:** Huan Weng, Wen Bo Tian, Zi Dong Xiao, Lin Xu

**Affiliations:** 1 Shantou Central Hospital Department of Endocrinology Shantou China Department of Endocrinology, Shantou Central Hospital, Shantou, China; 2 Sun Yat-sen University School of Public Health Guangzhou China School of Public Health, Sun Yat-sen University, Guangzhou, China

**Keywords:** Antithyroid agents, recurrence, Graves’ disease, risk factors, sleep initiation, maintenance disorders

## Abstract

**Objective:**

A common problem with antithyroid drugs (ATD) treatment in patients with Graves’ disease (GD) is the high recurrence rate after drug withdrawal. Identifying risk factors for recurrence is crucial in clinical practice. We hereby prospectively analyze risk factors for the recurrence of GD in patients treated with ATD in southern China.

**Subjects and methods:**

Patients who were newly diagnosed with GD and aged > 18 years were treated with ATD for 18 months and followed up for 1 year after ATD withdrawal. Recurrence of GD during follow-up was assessed. All data were analyzed by Cox regression with P values < 0.05 considered statistically significant.

**Results:**

A total of 127 Graves’ hyperthyroidism patients were included. During an average follow-up of 25.7 (standard deviation = 8.7) months, 55 (43%) had a recurrence within 1 year after withdraw of anti-thyroid drugs. After adjustment for potential confounding factors, the significant association remained for the presence of insomnia (hazard ratio (HR) 2.94, 95% confidence interval (CI) 1.47-5.88), greater goiter size (HR 3.34, 95% CI 1.11-10.07), higher thyrotrophin receptor antibody (TRAb) titer (HR 2.66, 95% CI 1.12-6.31) and a higher maintenance dose of methimazole (MMI) (HR 2.14, 95% CI 1.14-4.00).

**Conclusions:**

Besides conventional risk factors (i.e., goiter size, TRAb and maintenance MMI dose) for recurrent GD after ATD withdraw, insomnia was associated with a 3-fold risk of recurrence. Further clinical trials investigating the beneficial effect of improving sleep quality on prognosis of GD are warranted.

## INTRODUCTION

Graves’ disease (GD) is one of the organ-specific autoimmune diseases and the most common cause of hyperthyroidism ( 1 ). Treatment methods of GD mainly include radioactive iodine (I-131), surgery, and antithyroid drugs (ATD) ( 2 ). Two common ATD used in China include methimazole (MMI) and propylthiouracil, but they are, to some extent, limited by the risk of relapse after stopping the drug. It has been reported that the relapse rate is as high as 50% ( 3 ).

Previous studies suggested that factors affecting the recurrence of GD include male sex, smoking, presence of goiter, thyroid inferno and persistent high titer of thyroid stimulating hormone receptor antibody (TRAb) ( 4 – 6 ). Recently, there has been some evidence suggesting older age ( 7 ), mental disorder ( 8 ), and the use of iodized salt ( 9 ) were also related to a higher risk of GD recurrence. Moreover, maintenance dose of ATD treatment and use of levothyroxine therapy may also affect the recurrence of GD ( 10 ). However, there may be more factors affecting the recurrence of GD, and the effects of the reported factors may vary greatly by regions and populations. Hence, we conducted this prospective hospital-based study on newly diagnosed GD patients from southern China who regularly used ATD treatment to explore risk factors of GD relapse. Our study of an understudied population may provide clinical evidence for improving the prognosis of GD patients who underwent ATD treatment in southern China.

## SUBJECTS AND METHODS

### Statement of human rights and patient consent

The Shantou Central Hospital Ethics Committee approved the study and all participants gave written, informed consent before participation.

### Study population

**Figure 1:**
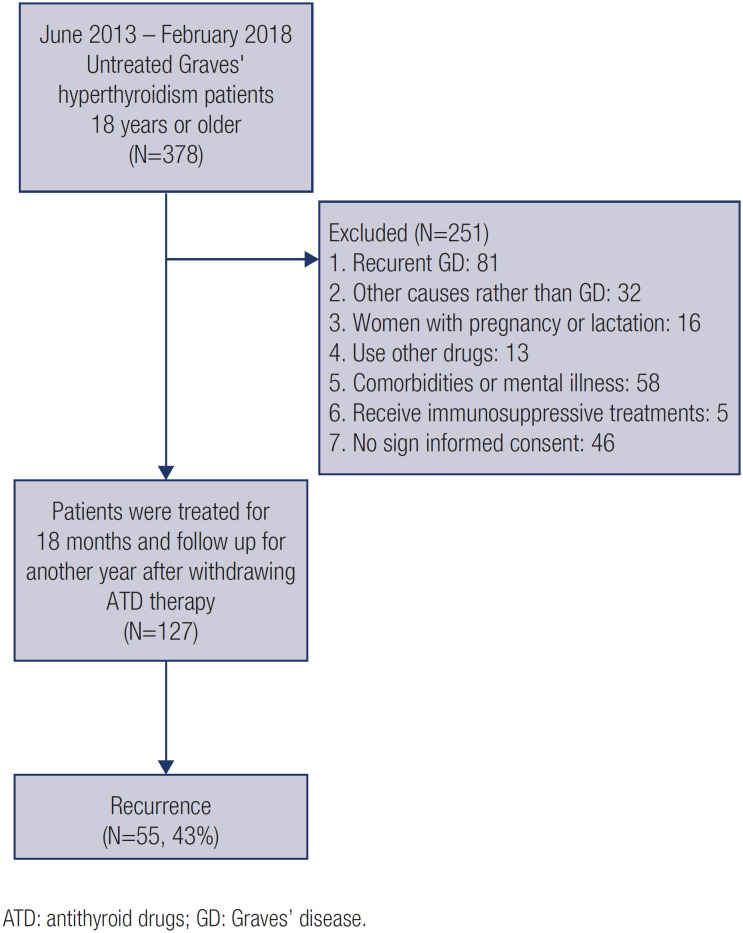
Flowchart of study.

We included consecutive untreated patients at the Shantou Central Hospital with a first episode of Graves’ hyperthyroidism from June 2013 to February 2018 in this study. Eligible patients were aged 18 years or old, local permanent residents and who had not been received antithyroid drugs, surgery or I-131 treatments before. The diagnosis of Graves’ disease hyperthyroidism includes the following ( 11 ): (1) presence of clinical symptoms and signs of hyperthyroidism; (2) diffuse thyroid enlargement as confirmed by palpation and ultrasound; (3) elevated serum total thyroxine 4 (TT4) and/or free thyroxine 4 (FT4), and reduced thyroid stimulating hormone (TSH); (4) exophthalmos or other eye signs; (5) anterior tibial mucinous edema; (6) TRAb positive. In addition, patients with the following conditions were excluded: (1) recurrent GD, including those who relapsed after ATD, I-131 or surgical treatment; (2) hyperthyroidism caused by other causes other than GD; (3) women with pregnancy or lactation; (4) concurrent use of drugs that might affect thyroid function (i.e., amiodarone or interferon); (5) presence of comorbidities including hyperthyroid heart disease or atrial fibrillation; (6) moderate to severe liver or renal dysfunction; (7) presence of psychiatric diseases or receiving chemotherapy or other immunosuppressive treatments; (8) patients who did not sign informed consent. All patients received ATD treatment after diagnosis. Patients were treated for 18 months and followed up for another year after withdrawing ATD therapy ( Figure 1 ).

### Risk factors and outcome

Information on demographic characteristics and medical history were collected during the clinical interview. Potential predictors considered in the regression model included age (per year), sex (female/male), family history of hyperthyroidism (yes/no), use of non-iodized salt (yes/no), current smoking status (yes/no), insomnia (yes/no), goiter size (0-I/II-III), thyroid volume (grade I & II/grade III), free triiodothyronine (FT3)/FT4 ratio, TSH, TRAb, thyroid inferno (yes/no), and maintenance dose of MMI (≤5/>5 mg/day). Patients were asked whether they suffered from insomnia when Graves’ disease hyperthyroidism was diagnosed. Insomnia was defined by having impaired daytime performance due to difficulty initiating sleep, difficulty maintaining sleep, or early morning awakening without ability to return to sleep. The diagnosis of insomnia was based on the third edition of the International Classification of Sleep Disorders (ICSD-3) ( 12 ). Thyroid inferno was defined as a large area of redness observed when the thyroid is examined by ultrasound. Recurrent hyperthyroidism was defined as a TSH less than 0.1 uIU/mL in combination with an elevated FT4 (>14.4 pmol/L) and/or FT3 (>6.0 pmol/L) ( 4 ).

### Statistical analysis

Categorical variables were described as frequency and percentages, and continuous variables as mean and standard deviation or median and interquartile range (IQR). Means for continuous variables were compared using one-way analysis of variance (ANOVA) when the data were normally distributed; otherwise, the Mann-Whitney test was used. Comparison of categorical variables was done using the χ^2^ test. Cox regression was used to explore factors associated with the recurrence of GD, giving hazards ratios (HRs) and 95% confidence intervals (CIs). All variables included in the multivariable regression were categorized as shown in Table 1 . All statistical analyses were performed using STATA/SE version 16.0 software (Stata Corp. 2019. Stata Statistical Software: Release 16. College Station, TX: Stata Corp LLC). A 2-sided α of less than 0.05 was considered statistically significant.

**Table 1:** Baseline characteristics of patients with a first episode of Graves’ hyperthyroidism before start of antithyroid drug therapy by status of recurrence

		Nonrecurrence	Recurrence	P-value
Number		72	55	-
Male sex, N (%)		20 (27.78)	12 (21.82)	0.44
Age, y, mean (SD)		39.6 (13.3)	35.2 (13.3)	0.07
Family history, yes, N (%)		27 (37.50)	17 (30.91)	0.44
Non-iodized salt, yes, N (%)		62 (86.11)	39 (70.91)	0.04
Current smoking, yes, N (%)		16 (22.22)	8 (14.55)	0.27
Insomnia, yes, N (%)		7 (9.72)	18 (32.73)	0.001
Goiter size, N (%)				<0.001
	0-I	26 (36.11)	4 (7.27)	
	II-III	46 (63.89)	51 (92.73)	
Thyroid volume, N (%)				
	Grade III	26 (36.11)	34 (61.82)	0.004
FT3/FT4 ratio, mean (SD)		0.5 (0.1)	0.6 (0.4)	0.15
TSH, uIU/mL, N (%)				0.55
	≤0.003	21 (29.17)	21 (38.18)	
	0.004-0.014	24 (33.33)	17 (30.91)	
	0.015-0.216	27 (37.50)	17 (30.91)	
TRAb, N (%)				<0.001
	1.82-12.00	31 (43.06)	11 (20.00)	
	12.01-24.99	27 (37.50)	15 (27.27)	
	≥25.00	14 (19.44)	29 (52.73)	
Thyroid inferno, yes, N (%)		43 (59,72)	52 (94.55)	<0.001
Graves’ ophthalmopathy, yes, N (%)		4 (5.56)	15 (27.27)	0.001
Maintenance dose of MMI, mg/d, N (%)				<0.001
	≤5	64 (88.89)	28 (50.91)	
	>5	8 (11.11)	27 (49.09)	
L-T4 therapy, yes, N (%)		12 (16.67)	12 (21.82)	0.46

FT3: free triiodothyronine; FT4: free thyroxine; L-T4: L-thyroxine; MMI, methimazole; SD: standard deviation; TRAb: thyrotrophin receptor antibody; TSH: thyroid stimulating hormone.

## RESULTS

A total of 127 consecutive newly diagnosed, untreated Graves’ hyperthyroidism patients were included. Of them, seven patients (5.5%) had been treated for less than 18 months (ranged from 9.2 to 14.3 months), and did not show recurrence after withdrawal of ATD. During an average follow-up of 25.7 (standard deviation = 8.7) months, 55 (43%) had a recurrence and 24 of them relapsed within 1 year after withdrawal of anti-thyroid drugs. Table 1 shows that at baseline, patients who had a recurrence had lower proportion of using non-iodized salt and higher proportion of insomnia (P from 0.001 to 0.04). Moreover, patients who had a recurrence had greater goiter size and thyroid volume, higher TRAb titer and prevalence of thyroid inferno and Graves’ ophthalmopathy, and higher maintenance dose of MMI (all P from <0.001 to 0.004). No differences were found for sex, age, family history of hyperthyroidism, smoking, FT3/FT4 ratio, TSH and the use of L-T4 therapy (all P > 0.05).

In the univariate analyses, the presence of insomnia (HR 2.52, 95% CI 1.43-4.45), greater goiter size (HR 4.85, 95% CI 1.75-13.43), greater thyroid volume (HR 2.06, 95% CI 1.19-3.55), higher TRAb titer (3.40, 95% CI 1.69-6.85), presence of thyroid inferno (7.37, 95% CI 2.30-23.64) and Graves’ ophthalmopathy (2.85, 95% CI 1.56-5.19), and a higher maintenance dose of MMI (3.38, 95% CI 1.98-5.78) were associated with a higher risk of recurrent GD. After mutual adjustment, the significant association remained for the presence of insomnia (HR 2.94, 95% CI 1.47-5.88), greater goiter size (HR 3.34, 95% CI 1.11-10.07), higher TRAb titer (HR 2.66, 95% CI 1.12-6.31) and a higher maintenance dose of MMI (HR 2.14, 95% CI 1.14-4.00) ( Table 2 ).

**Table 2:** Prediction of recurrence by baseline characteristics

		Crude HR (95% CI)	Adjusted HR (95% CI)
Male sex		0.83 (0.44-1.57)	0.70 (0.30-1.66)
Age		0.98 (0.96-1.00)	0.99 (0.97-1.01)
Family history, yes		0.83 (0.47-1.46)	0.98 (0.53-1.82)
Non-iodized salt, yes		0.57 (0.32-1.03)	0.83 (0.42-1.62)
Current smoking, yes		0.69 (0.33-1.46)	0.68 (0.24-1.92)
Insomnia, yes		2.52 (1.43-4.45) ***	2.94 (1.47-5.88) **
Goiter size			
	0-I	1.00	1.00
	II-III	4.85 (1.75-13.43) **	3.34 (1.11-10.07) *
Thyroid volume			
	Grade I & II	1.00	1.00
	Grade III	2.06 (1.19-3.55) *	1.31 (0.69-2.5)
FT3/FT4 ratio		1.52 (0.81-2.86)	0.80 (0.32-1.96)
TSH, uIU/mL			
	≤0.003	1.00	1.00
	0.004-0.014	1.35 (0.71-2.56)	0.82 (0.41-1.66)
	0.015-0.216	1.06 (0.54-2.08)	1.00 (0.48-2.10)
TRAb			
	1.82-12.00	1.00	1.00
	12.01-24.99	1.48 (0.68-3.22)	1.24 (0.50-3.06)
	≥25.00	3.40 (1.69-6.85) **	2.66 (1.12-6.31) *
Thyroid inferno		7.37 (2.30-23.64) **	3.04 (0.84-10.97)
Graves’ ophthalmopathy		2.85 (1.56-5.19) ***	1.85 (0.93-3.67)
Maintenance dose of MMI, mg/d			
	≤5	1.00	1.00
	>5	3.38 (1.98-5.78) ***	2.14 (1.14-4.00) *
L-T4 therapy		1.19 (0.63-2.26)	1.01 (0.48-2.12)

CI: confidence interval; FT3: free triiodothyronine; FT4: free thyroxine; HR: hazard ratio; L-T4: L-thyroxine; MMI: methimazole; TRAb: thyrotrophin receptor antibody; TSH: thyroid stimulating hormone.

* P < 0.05

** P < 0.01

*** P < 0.001.

**Figure 2:**
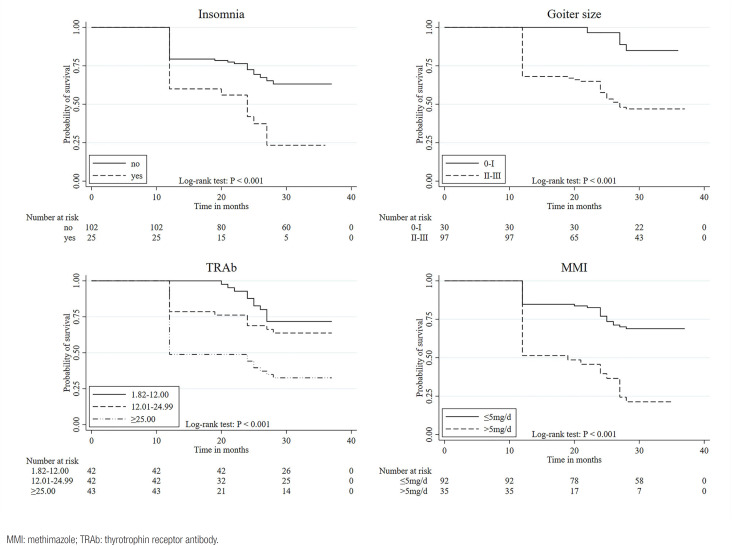
Kaplan-Meier survival estimates according to insomnia (A), goiter size (B), TRAb groups (C) and different maintenance MMI doses (D).

Kaplan-Meier survival estimates shows that patients who had insomnia, greater goiter size and higher TRAb titer at GD onset, and higher maintenance dose of MMI had lower probability of “survival” from recurrence ( Figure 2 ).

## DISCUSSION

In this understudied population, we showed that the presence of insomnia, greater goiter size and higher TRAb titer at the onset of GD, as well as higher maintenance dose of MMI were associated with the recurrence of GD after drug withdraw. Our results were consistent with a recent systematic review and meta-analysis ( 5 ) and have extended the findings of this meta-analysis by indicating that patients with self-reported insomnia had almost 3-fold risk of recurrence in GD patients after ATD withdraw. Although the exact mechanism is unclear, the results indicate that the restorative effect of sleep is important in the neuro-immunologic function of an individual.

The recurrence of GD after ATD withdraw could be affected by some factors, such as female gender, age, family history, mental stress, smoking, thyroid volume, thyroid inferno, Graves’ ophthalmopathy, TRAb, TSH and FT3/FT4 ratio ( 5 ). Mental factors such as stress or depression may be involved in the pathogenesis of GD and play an important role in the occurrence and development of the disease ( 13 , 14 ). Previous studies have shown that GD patients who had been exposed to stress had poorer prognosis ( 13 , 14 ). A prospective study of 300 GD patients showed that positive coping styles and social support were associated with recovery from GD patients ( 15 ). Our results, together with previous findings of others, support that insomnia may be a risk factor for relapse in GD patients who had withdrawn ATD treatment.

In our study, patients with a smaller goiter size had a significantly lower risk of recurrence after treatment withdraw. No matter how the goiter size was determined (i.e., by physician's palpation or measured by ultrasound), its size at the first diagnosis of GD or its change at the end of ATD treatment would have a great predictive function for recurrence in GD patients after ATD withdrawal. A previous study showed that after a 5-year follow-up of GD patients with different thyroid volumes at the initial onset, patients with no or only mild thyroid enlargement before treatment had a lower risk for long-term remission rate than patients with moderate to severe thyroid enlargement ( 3 ). Another prospective study of 2,405 GD patients showed that patients with goiter at the first onset tend to be more severely ill ( 6 ). Furthermore, a study in China also showed that the 2-year remission rate of Graves’ disease after antithyroid drug withdraw was significantly higher in patients with larger goiter than those with normal or mildly enlarged thyroid ( 16 ).

Previous studies have shown that the onset of GD is associated with the competitive binding of stimulating antibodies in TRAb with TSH receptors in thyroid tissue ( 17 ). The activity of TRAb increases during the onset of GD and decreases during the remission phase ( 18 , 19 ), and might increase again during recurrence, suggesting that TRAb may be an indicator for monitoring the efficacy of the treatment of GD ( 20 ). However, the value of TRAb in predicting clinical outcomes of ATD therapy has been controversial. In some patients who had recurrence of hyperthyroidism after ATD withdrawal, the TRAb titer remained high ( 21 ). This was also indicated by a study in mice showing that excessive autoantibody stimulation reduces the sensitivity of the thyroid gland to TRAb through downregulating TSH receptors ( 22 ). This might be due to a change in thyroid gland responsiveness to TRAb, which may be caused by the progression of cell-mediated autoimmune destruction during the disease process of GD ( 18 ). Furthermore, the nature of the antibody might be changed after long-term ATD therapy. Although the TRAb levels remained, they could be functionally different and unmeasurable in serum for clinical or biochemical hyperthyroidism ( 23 ), because the production of TRAb is confined to the thyroid gland and adjacent lymph nodes without spill-over of antibodies into the circulation ( 24 ). However, our results demonstrated that TRAb at diagnosis significantly predicted the recurrence after ATD withdraw, which supports the important role of TRAb at diagnosis in risk classification of GD patients.

Maintenance dose of ATD is closely related to the prognosis of GD. Previous studies suggest that the use of higher doses of drugs may be of benefit. However, this idea has turned out to be misguided, because higher doses of ATD lead to more side effects of the drugs ( 25 , 26 ). To minimize the risk of side effects, the lowest possible dose of the drug should be used, and such therapy may prevent recurrence of overt GD ( 27 ). One mechanism underlying such a protective effect of low dose MMI therapy may be a decrease in the risk of reactivation of the vicious cycle. Another possibility would be that the risk of recurrence is reduced by keeping thyroid iodine content low ( 28 ). Furthermore, we found no significant influence of sex or smoking in GD recurrence predisposition, possibly due to the relatively small number of patients in our study.

There are some potential limitations to the current study. We did not have information on socioeconomic position or personal lifestyle factors such as income, occupation, daily habitual activities, and records of physical activity. The potential impact of lacking these potential confounders in Cox models on the risk of recurrence, although unclear, cannot be large enough to alter the direction of the overall risk estimation. Another potential limitation is the lack of covariate analyses of medication use, particularly the type of sleep-inducing pills prescribed and concurrent medications taken, which were too complex to be incorporated into this study. In addition, it is not possible to ascertain whether the patients actually took the medications prescribed to them. Also, we did not have data to explore whether the concurrent use of other medication or presence of co-morbidities might have influenced or modified the results. Moreover, we did not collect information on peak systolic velocity of thyroid artery and only assessed the presence of thyroid inferno. Since this is a grade-A Tertiary Hospital in the southern China, all radiologists are well-trained. Thus, the misclassification, if any, should be minimized. In addition, information on quality of life was not collected. Thus, the effect of Graves’ disease on patients’ the quality of life as well as their recurrence warrants further studies. Finally, as this is an observational study, causal associations between identified predictors and the recurrence of GD cannot be established. For some modifiable factors, such as insomnia, further interventional studies examining whether the use of sleep-inducing drugs in GD patients with insomnia reduces relapse rate are needed to establish causal links.

In conclusion, besides conventional risk factors (i.e., goiter size, TRAb and maintenance MMI dose) for recurrent GD after ATD withdraw, insomnia was associated with a 3-fold risk of recurrence. Further clinical trials investigating the beneficial effect of improving sleep quality on prognosis of GD are warranted.
